# Rotation Estimation: A Closed-Form Solution Using Spherical Moments †

**DOI:** 10.3390/s19224958

**Published:** 2019-11-14

**Authors:** Hicham Hadj-Abdelkader, Omar Tahri, Houssem-Eddine Benseddik

**Affiliations:** 1 IBISC lab. EA 4526, University of Evry-Val-d’Essonne-Paris Saclay, 91000 Evry, France; 2 INSA Centre Val de Loire, PRISME lab. EA 4229, University of Orleans, 18000 Bourges, France; 3 MIS lab. EA 4290, University of Picardie Jules Verne, 80039 Amiens, France; houssem.eln@gmail.com

**Keywords:** geometric moments, spherical image, motion estimation

## Abstract

Photometric moments are global descriptors of an image that can be used to recover motion information. This paper uses spherical photometric moments for a closed form estimation of 3D rotations from images. Since the used descriptors are global and not of the geometrical kind, they allow to avoid image processing as features extraction, matching, and tracking. The proposed scheme based on spherical projection can be used for the different vision sensors obeying the central unified model: conventional, fisheye, and catadioptric. Experimental results using both synthetic data and real images in different scenarios are provided to show the efficiency of the proposed method.

## 1. Introduction

Rotation estimation from images is important for many application as for motion estimation and registration in image processing [1], pattern recognition [2], localization and control of ground/aerial vehicles [3], and computer vision [4]. Feature based and direct (appearance based) are the two main approach categories proposed in the literature. The first method type uses Epipolar geometry applied to geometrical features as as points [5], lines [6], or contours [7] to estimate the fundamental matrix (uncalibrated vision sensors), the essential matrix, or homography matrix for the calibrated case. Examples of such methods using conventional cameras are Agrawal et al. [8] and Malis et al. [9] and using omnidirectional vision sensors is [10,11,12]. The direct approaches, on the other hand, avoid geometric features extraction by using grey level information of the whole image to define the descriptors to be used. Those methods are referred to in the literature as dense, direct, or global. Methods within this category can avoid the need of some higher-level image processing steps such as feature extraction, matching, and tracking. For instance, Reference [13] exploits optical flow estimation. However, a consequence of using optical flow in References [13,14] is to be limited to small camera displacements. To deal with larger motion, in Reference [4], Makadia and Daniilidis proposed a method using correlations between the two sets of spherical harmonic coefficients.

To address even larger camera displacements, motion estimation techniques based on global image representation is utilized in the literature. Makadia and Daniilidis [4] represented the spherical omnidirectional images in the frequency domain to recover rotation using the maximum correlation between the two sets of spherical harmonic coefficients. In Reference [15], the authors proposed to estimate the rotation based on 3D-mesh representation of spherical image intensities. However, these methods are not often computationally efficient due to the computation load of spherical harmonic coefficients.

The moments as descriptors provide useful characteristics such as the center of gravity of an object, orientation, disparity, and volume. As Fourier descriptors, they can be used as a compact representation of the image. In the case of 2D images, in addition to the classical geometrical moments, other kinds of moments have been defined in the literature as Zernike Moments [16] or complex moments [17]. Invariant characteristics to some transformations that an object can undergo, commonly called moment invariants, found many applications such as for pattern recognition. Thanks to their decoupling properties, they also have been used to define a minimal number of features for visual servoing and pose estimation as in References [18,19] and for visual servoing as in References [20,21].

Spherical projections can be obtained from images if the used camera obeys the unified central model. By using such projections, the authors in Reference [22] proposed a decoupled visual servoing scheme using spherical moments computed for a set of matched points. The proposed features allow to control the translational motions independently from the rotational ones. The current work exploits the spherical projection to estimate 3D rotations using spherical moments. The proposed scheme provides an analytical solution to rotation. An early version of this work was published in Reference [23]. In the next section, the central camera model and the definition of spherical moments are first recalled. The proposed approach is subsequently presented. In Section 3, experimental results of rotation estimation using synthetic data and real images acquired from a catadioptric camera mounted on a manipulator robot (Staubli) and a mobile robot (Pioneer) are presented to validate our approach.

## 2. Direct Estimation of Rotation Using Spherical Moments

### 2.1. Spherical Moments

The projection onto a unitary sphere t $P_{s}={\left[x_{s}\;\;\;\;y_{s}\;\;\;\;z_{s}\right]}^{⊤}$ of a 3D point P is defined by the following:

(1) $$P_{s}={\left[x_{s}\;\;\;\;y_{s}\;\;\;\;z_{s}\right]}^{⊤}=\frac{P}{\left\|P\right\|}.$$

Actually, the projection onto the unit sphere can be obtained using the unified central model for conventional, fisheye, or catadioptric [24]. This makes the proposed scheme in this work valid for a wide range of cameras.

From a spherical image (or Gaussian sphere [25]) of the environment, the photometric moments can be defined by the following:

(2) $$m_{ijk}={\;\int \int \;}_{S}x_{s}^{i}\;\;y_{s}^{j}\;\;z_{s}^{k}\;\;I\left(x_{s},y_{s},z_{s},t\right)\;ds,$$

where *S* is defined as the sphere surface. Let R be a rotation matrix and ω be the instantaneous rotational speed applied to the sensor frame. First, it can be easily shown that a rotational motion defined by a matrix R on 3D point P ($P^{\prime }=R\;P$, where $P^{\prime }$ is the same 3D point after rotation) induces the same motion on its projection onto the unit sphere ($P_{s}^{\prime }=R\;P_{s}$). In tangent space, the effect of a rotation speed $\omega ={\left[\omega_{x}\;\;\;\omega_{y}\;\;\;\omega_{z}\right]}^{⊤}$ on a projected point on the sphere is given by its time derivative ${\dot{P}}_{s}$:

(3) $${\dot{P}}_{s}={\left[P_{s}\right]}_{\times }\omega =-{\left[\omega \right]}_{\times }P_{s}=L_{P_{s}}\;\omega =\left[\begin{array}{ccc} 0 & -z_{s} & y_{s}\\ z_{s} & 0 & -x_{s}\\ -y_{s} & x_{s} & 0 \end{array}\right]\omega ,$$

where $L_{P_{s}}$ is the interaction matrix and ${\left[\;\;\;\right]}_{\times }$ defines the skew-symmetric matrix of a vector. On the other side, by taking the time derivative of Equation (2), the time variation of the moment $m_{ijk}$ is linked to rotational speed ω by the following [26]:

(4) $${\dot{m}}_{ijk}={\left[\begin{array}{c} jm_{i,j-1,k+1}-km_{i,j+1,k-1}\\ km_{i+1,j,k-1}-im_{i-1j,k+1}\\ im_{i-1,j+1,k}-jm_{i+1,j-1,k} \end{array}\right]}^{⊤}\omega .$$

In the following, a scheme is proposed to define triples $x_{m}={\left[x\left(m_{ijk}\right)\;\;\;y\left(m_{ijk}\right)\;\;\;z\left(m_{ijk}\right)\right]}^{⊤}\in R^{3\times 1}$ that behave as a projected point onto the sphere with respect to rotations.

### 2.2. Closed-Form Solution of Rotation Estimation

From moments of order 1 and by using Equation (4), the interaction matrix that links time derivatives of the triple $x_{m_{1}}={\left[m_{100}\;\;\;m_{010}\;\;\;m_{001}\right]}^{⊤}$ to the rotational velocities is obtained by the following:

(5) $${\dot{x}}_{m_{1}}=\left[\begin{array}{ccc} 0 & -m_{001} & m_{010}\\ m_{001} & 0 & -m_{100}\\ -m_{010} & m_{100} & 0 \end{array}\right]\omega ={\left[x_{m_{1}}\right]}_{\times }\omega .$$

As it can be seen, the interaction matrix given by Equation (5) has an identical form to the one obtained for a projected point onto the sphere. This implies a 3D rotation results in the same motion on $x_{m_{1}}$. Let us now consider triples $x_{m}$ defined from moments of orders larger than 1. In order to have $x_{m}^{\prime }=R\;x_{m}$, the interaction matrix related to $x_{m}={\left[x\left(m_{ijk}\right),\;\;y\left(m_{ijk}\right),\;\;z\left(m_{ijk}\right)\right]}^{⊤}$ must have the same form as Equation (3), which means the following:

(6) $$\begin{array}{cc} {\dot{x}}_{m} & =\left[\begin{array}{ccc} 0 & -z(m_{ijk}) & y(m_{ijk})\\ z(m_{ijk}) & 0 & -x(m_{ijk})\\ -y(m_{ijk}) & x(m_{ijk}) & 0 \end{array}\right]\omega =-{\left[\omega \right]}_{\times }x_{m} \end{array}$$

Under the assumption of constant speed (i.e., ${\left[\omega \right]}_{\times }$ is a constant matrix), solving for Equation (6) leads to the following:

(7) $$x_{m}\left(t\right)=exp\left(-{\left[\omega \right]}_{\times }t\right)x_{m}\left(0\right)=Rx_{m}\left(0\right)$$

where $x_{m}\left(0\right)$ and $x_{m}\left(t\right)$ are respectively the initial value and at the value time *t* of the triple (which defines the relation for a couple of images). Any rotational motion between two images can be obtained by applying a constant speed during time *t*. This means Equation (6) implies $x_{m}\left(t\right)=Rx_{m}\left(0\right)$ for any couple of images.

Let us now explain the procedure to obtain those triples using as an example: the moment vector $w_{23}={\left[m_{300}m_{200}\;\;\;m_{300}m_{110}\;\;\;\cdots \;\;\;m_{003}m_{002}\right]}^{⊤}$. The latter is composed of all the possible products between moments of order 2 and those of order 3. There are 6 possible moments of order equal to 2 and 10 of order equal to 3. This implies that $w_{23}$ is of size equal to 60. Let us define triples $x_{w_{23}}={\left[\alpha_{x}^{⊤}w_{23}\;\;\;\;\;\;\;\alpha_{y}^{⊤}w_{23}\;\;\;\;\;\;\alpha_{z}^{⊤}w_{23}\right]}^{⊤}$, where $\alpha_{x}$, $\alpha_{y}$, and $\alpha_{z}$ are vectors of coefficients that define the triple coordinates as linear combinations of the entries of $w_{23}$. Our goal is to find the conditions on those vectors of coefficients such that the time derivative ${\dot{x}}_{w_{23}}$ is of the same form as Equation (6).

As an example, let us consider the time derivative of the moment product $m_{030}m_{200}$, which is a component of $w_{23}$. Using Equation (4), we have the following:

(8) $$\left\{\begin{array}{c} {\dot{m}}_{200}=0\times \omega_{x}-2m_{101}\omega_{y}+2m_{110}\omega_{z}\\ {\dot{m}}_{030}=3m_{021}\omega_{x}+0\times \omega_{y}-3m_{120}\omega_{z} \end{array}\right..$$

As it can be also concluded from Equation (4), the time variation of a moment is a function of moments of the same order and the rotational speed. From Equation (8), we obtain the time derivative of the product $m_{030}m_{200}$ by the following:

(9) $$\begin{array}{cc} \; & \frac{d(m_{030}m_{200})}{dt}=m_{200}{\dot{m}}_{030}+m_{030}{\dot{m}}_{200}\\ \; & =3m_{200}m_{021}\omega_{x}-2m_{101}m_{030}\omega_{y}+\left(2m_{110}m_{030}-3m_{200}m_{120}\right)\omega_{z}. \end{array}$$

Note that the coefficients of $\omega_{x}$, $\omega_{y}$, and $\omega_{z}$ are nothing but linear combinations of products between moments of orders 2 and 3. Actually, the time derivative of any component of the vector $w_{23}$ will be a linear combination of the other components. Based on this fact, ${\dot{w}}_{23}$ can be written under the following form:

(10) $${\dot{w}}_{23}=\left(L_{w_{23}/\omega_{x}}\;\;w_{23}\right)\omega_{x}+\left(L_{w_{23}/\omega_{y}}\;\;w_{23}\right)\omega_{y}+\left(L_{w_{23}/\omega_{z}}\;\;w_{23}\right)\omega_{z},$$

where $L_{w_{23}/\omega_{x}}$, $L_{w_{23}/\omega_{y}}$ and $L_{w_{23}/\omega_{z}}$ are 60×60 matrices. Using Equation (10), the time derivative of the triple $x_{w_{23}}={\left[\alpha_{x}^{⊤}w_{23}\;\;\;\;\;\;\;\alpha_{y}^{⊤}w_{23}\;\;\;\;\;\;\alpha_{z}^{⊤}w_{23}\right]}^{⊤}$ is obtained:

(11) $${\dot{x}}_{w_{23}}=\left[\begin{array}{ccc} \alpha_{x}^{⊤}L_{w_{23}/\omega_{x}}w_{23} & \alpha_{x}^{⊤}L_{w_{23}/\omega_{y}}w_{23} & \alpha_{x}^{⊤}L_{w_{23}/\omega_{z}}w_{23}\\ \alpha_{y}^{⊤}L_{w_{23}/\omega_{x}}w_{23} & \alpha_{y}^{⊤}L_{w_{23}/\omega_{y}}w_{23} & \alpha_{y}^{⊤}L_{w_{23}/\omega_{z}}w_{23}\\ \alpha_{z}^{⊤}L_{w_{23}/\omega_{x}}w_{23} & \alpha_{z}^{⊤}L_{w_{23}/\omega_{y}}w_{23} & \alpha_{z}^{⊤}L_{w_{23}/\omega_{z}}w_{23} \end{array}\right]\omega .$$

If Equation (11) is of the same form as Equation (6), this leads to the 9 following conditions:

(12) $$\begin{array}{cc} \;\begin{array}{cc} \; & 1)\alpha_{x}^{⊤}L_{w_{23}/\omega_{x}}w_{23}\;=\;0;\\ \; & 2)\alpha_{x}^{⊤}L_{w_{23}/\omega_{y}}w_{23}\;=\;-\alpha_{z}^{⊤}w_{23};\\ \; & 3)\alpha_{x}^{⊤}L_{w_{23}/\omega_{z}}w_{23}\;=\;\alpha_{y}^{⊤}w_{23};\\ \; & 4)\alpha_{y}^{⊤}L_{w_{23}/\omega_{x}}w_{23}\;=\;\alpha_{z}^{⊤}w_{23};\\ & 5)\alpha_{y}^{⊤}L_{w_{23}/\omega_{y}}w_{23}\;=\;0; \end{array}\;\; & \begin{array}{cc} \; & 6)\alpha_{y}^{⊤}L_{w_{23}/\omega_{z}}w_{23}\;=\;-\alpha_{x}^{⊤}w_{23};\\ \; & 7)\alpha_{z}^{⊤}L_{w_{23}/\omega_{x}}w_{23}\;=\;-\alpha_{y}^{⊤}w_{23};\\ \; & 8)\alpha_{z}^{⊤}L_{w_{23}/\omega_{y}}w_{23}\;=\;\alpha_{x}^{⊤}w_{23};\\ \; & 9)\alpha_{z}^{⊤}L_{w_{23}/\omega_{z}}w_{23}\;=\;0. \end{array} \end{array}$$

The 9 constraints given by Equation (12) must be valid for any value of $w_{23}$. Therefore, they can be simplified as follows:

(13) $$\;\begin{array}{ccc} \begin{array}{cc} \; & 1)\alpha_{x}^{⊤}L_{w_{23}/\omega_{x}}\;=\;0\\ \; & 2)\alpha_{x}^{⊤}L_{w_{23}/\omega_{y}}\;=\;-\alpha_{z}^{⊤}\\ \; & 3)\alpha_{x}^{⊤}L_{w_{23}/\omega_{z}}\;=\;\alpha_{y}^{⊤} \end{array}\; & \;\begin{array}{cc} \; & 4)\alpha_{y}^{⊤}L_{w_{23}/\omega_{x}}\;=\;\alpha_{z}^{⊤}\\ \; & 5)\alpha_{y}^{⊤}L_{w_{23}/\omega_{y}}\;=\;0\\ \; & 6)\alpha_{y}^{⊤}L_{w_{23}/\omega_{z}}\;=\;\;-\;\alpha_{x}^{⊤} \end{array}\; & \;\begin{array}{cc} \; & 7)\alpha_{z}^{⊤}L_{w_{23}/\omega_{x}}\;=\;\;-\;\alpha_{y}^{⊤}\\ \; & 8)\alpha_{z}^{⊤}L_{w_{23}/\omega_{y}}\;=\;\alpha_{x}^{⊤}\\ \; & 9)\alpha_{z}^{⊤}L_{w_{23}/\omega_{z}}\;=\;0. \end{array} \end{array}$$

The constraint set of Equation (13) can be written under the following form:

(14) $$\left[\begin{array}{ccc} L_{w_{23}/\omega_{x}}^{⊤} & 0 & 0\\ L_{w_{23}/\omega_{y}}^{⊤} & 0 & I\\ L_{w_{23}/\omega_{z}}^{⊤} & -I & 0\\ \vdots & \vdots & \vdots \\ 0 & 0 & L_{w_{23}/\omega_{z}}^{⊤} \end{array}\right]\left[\begin{array}{c} \alpha_{x}\\ \alpha_{y}\\ \alpha_{z} \end{array}\right]=0,$$

where I and 0 are respectively the identity and zero matrices of size 60×60. In practice, $L_{w_{23}/\omega_{x}}$, $L_{w_{23}/\omega_{y}}$ and $L_{w_{23}/\omega_{z}}$ are very sparse integer matrices. Solving Equation (14) for the case of the moment vector $w_{23}$ leads to three solutions for the triple $x_{w_{23}}$ (refer to Equations (A1)–(A3) in the Appendix A). Such conditions can be solved using a symbolic computation software as Matlab Symbolic Toolbox.

The procedure used to obtain triples from $w_{23}$ can be extended straightforwardly to a different moment vector as follows:

- Define the moment vector w similarly as for $w_{23}$. The vector can be built by moment products of two different orders or more. For instance, products such as $m_{200}m_{220}m_{300}$ which combine moments of order 2, 3, and 4 can be also used. the vector w has to be built from moment products of the same nature and has to also include all of them.
- Using Equation (4), compute the matrices $L_{w_{23}/\omega_{x}}$, $L_{w_{23}/\omega_{y}}$, and $L_{w_{23}/\omega_{z}}$ such that we obtain the following:
  (15) $$\dot{w}=\left(L_{w_{23}/\omega_{x}}\;w\right)\omega_{x}+\left(L_{w_{23}/\omega_{y}}\;w\right)\omega_{y}+\left(L_{w_{23}/\omega_{z}}\;w\right)\omega_{z}.$$
- Solve the following system:
  (16) $$\left[\begin{array}{ccc} L_{w_{23}/\omega_{x}}^{⊤} & 0 & 0\\ L_{w_{23}/\omega_{y}}^{⊤} & 0 & I\\ L_{w_{23}/\omega_{z}}^{⊤} & -I & 0\\ \vdots & \vdots & \vdots \\ 0 & 0 & L_{w_{23}/\omega_{z}}^{⊤} \end{array}\right]\left[\begin{array}{c} \alpha_{x}\\ \alpha_{y}\\ \alpha_{z} \end{array}\right]=0,$$
  where I and 0 are respectively the identity and zero matrices of the same size as $L_{w_{23}/\omega_{x}}$, $L_{w_{23}/\omega_{y}}$ and $L_{w_{23}/\omega_{z}}$.

In practice, two different triples $x_{w}$ are enough to obtain a closed-form solution of the rotational motion since they can define an orthonormal basis. Let us consider, for instance, the two triples $P_{1}$ and $P_{2}$ computed from the moments of a first image using Equations (A1) and (A2) and $P_{1}^{\prime }$ and $P_{2}^{\prime }$, their corresponding ones, computed from the image after rotation. Therefore, we have $P_{1}^{\prime }=RP_{1}$ and $P_{2}^{\prime }=RP_{2}$, where R is the rotation matrix. Let us define the following base:

(17) $$v_{1}=\frac{P_{n1}+P_{n2}}{\|P_{n1}+P_{n2}\|},\;\;v_{2}=\frac{P_{n1}-P_{n2}}{\|P_{n1}-P_{n2}\|},\;\;v_{3}=v_{1}\times v_{2}$$

where $P_{n1}=\frac{P_{1}}{\|P_{1}\|}$ and $P_{n2}=\frac{P_{2}}{\|P_{2}\|}$. First, it can be shown that the vectors $v_{1}$, $v_{2}$ and $v_{3}$ form an orthonormal base (i.e., $\|v_{1}\left\|=\right\|v_{2}\left\|=\right\|v_{3}\|=1$ and $v_{1}.v_{2}=v_{1}.v_{3}=v_{3}.v_{2}=0$). Second, let us now consider a second base ${v_{1}}^{\prime }$, $v_{2}^{\prime }$ and $v_{3}^{\prime }$ computed from $P_{1}^{\prime }$ and $P_{2}^{\prime }$ using Equation (17). Since $P_{1}^{\prime }=RP_{1}$ and $P_{2}^{\prime }=RP_{2}$, it can be proven using Equation (17) that $v_{1}^{\prime }=Rv_{1}$, $v_{2}^{\prime }=Rv_{2}$ and $v_{3}^{\prime }=Rv_{3}$. The last equations can be written under the following form:

(18) $$V=RV^{\prime },$$

where $V=[v_{1}\;\;v_{2}\;\;v_{3}]$ and $V^{\prime }=\left[v_{1}^{\prime }\;\;v_{2}^{\prime }\;\;v_{3}^{\prime }\right]$. Finally, we obtain:

(19) $$R=V\;\;{V^{\prime }}^{⊤},$$

where the rotation matrix can be estimated straightforwardly. Solving Equation (16) ensures that all possible triples are obtained from a given moment vector. Obtaining a mathematical proof on the existence and their number is out of the the scope of this contribution.

### 2.3. Rotation Estimation and Scene Symmetry

As explained in the previous paragraph, an orthonormal base can be built from the triples $P_{1}$ and $P_{2}$ to estimate the rotation matrix. In practice, if the scene has no symmetry, extensive tests (not included in the paper for brevity) show that using the triples $P_{1}$ and $P_{2}$ could be enough. If the scene has a symmetry with respect to a plane (for instance xy plane), the z-coordinates of the triples $P_{i}$ are null. This means that all $P_{i}$ will belong to the xy plane. If the scene is symmetrical with respect to xy and xz planes, for instance, then the triples $P_{i}$ will form a line in the direction of the x-axis.

## 3. Validation Results

In the following section, simulation results and real experiments are shown. For all the validation results, the spherical moments are computed directly using the pixel’s grey levels without warping the images to the sphere space. The used formulas are given in References [26,27] for the perspective and fisheye cases respectively:

(20) $$m_{ijk}={\;\int \int \;}_{\mathrm{Image}}x_{s}^{i}\;\;y_{s}^{j}\;\;z_{s}^{k}\;\;\frac{{\left(\xi +z_{s}\right)}^{3}}{1+\xi z_{z}}I\left(x,y,t\right)\;dxdy,$$

where ξ is the distortion parameter and (x,y) are the coordinates of a point in the metric image. The formulas of $x_{s}$, $y_{s}$, and $z_{s}$ as functions of (x,y) can be found in Reference [28]. Using Matlab with a computer equipped with a processor Intel(R) Core(TM) i5-7600 CPU @ 3.50 GHz 3.50 GHz and 16,0 Go RAM, the whole process including the computation of spherical moments of an image of size 480×640 pixels until the rotation estimation takes around 20 ms. This leaves room for reducing the time cost using other programming tools than Matlab to few milliseconds.

### 3.1. Simulation Results

The objective of this part is threefold:

- To show the validity of the proposed method to cameras obeying unified model, two different kinds of camera model are used to compute the images. The first corresponds to a simulated fisheye camera with the parameters chosen as focal scaling factors Fx=Fy=960 pixels, coordinates of the principal point $u_{x}=240$ and $u_{y}=320$ pixels, and distortion parameter ξ=1.6. The second model corresponds a conventional camera with focal scaling factors Fx=Fy=600 pixels and coordinates of the principal point $u_{x}=240$ and $u_{y}=320$;
- To Validate our approach for large rotational motion;
- To test the effect of translational motion on the accuracy of the estimated rotations.

The Matlab code used for those simulations results can be downloaded online (http://aramis.iup.univ-evry.fr:8080/~hadj-abdelkader/Sensors/Sim_ForSensors.zip).

**Figure 1:**
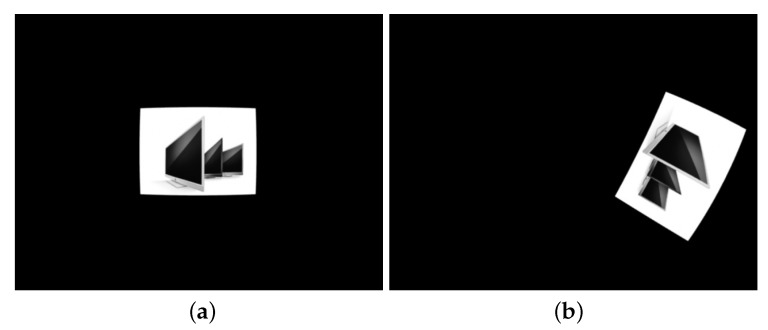
Results using a fisheye camera model: (**a**) the reference image and (**b**) example of rotated images.

**Figure 2:**
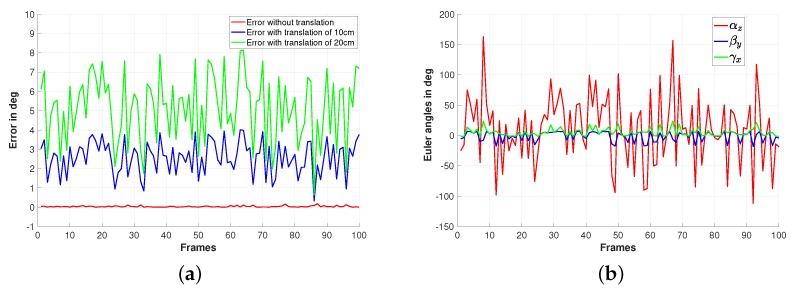
Simulation results using a fisheye camera model: (**a**) Estimation error of the rotation and (**b**) rotation ground truth expressed by Euler angles.

In the first results, a fisheye camera model is used to generate the images. More precisely, from the reference image shown on Figure 1a, three sets of 100 new images are computed based on randomly generated displacements. An example of the used images is shown on Figure 1b. All the three image sets were obtained with the same random rotations shown on Figure 2b but with translational motion of norms equal to 0 for the first set and 10 cm and 20 cm for the two others. For the two last sets, the same random translation directions are used but with different norms equal to 10 cm and 20 cm, respectively. To assess the estimated rotations, the norm of the vector composed by the errors on those Euler angles is used. The obtained accuracy results are shown on Figure 2a. It can be seen that, for the case where the camera displacement does not include translational motion, the rotation motion is well estimated (refer to the red plot of the Figure). The results obtained in the case where the camera displacement involves translational motion of norm 10 cm and 20 cm are shown respectively by the blue and the green plots of the Figure 2a. From the obtained plots, it can be seen that a maximum error of $4^{\circ }$ (respectively $8^{\circ }$) and an average error less then $3^{\circ }$ (respectively $5^{\circ }$) are obtained in the case of translation norm equal to 10 cm (respectively 20 cm).

**Figure 3:**
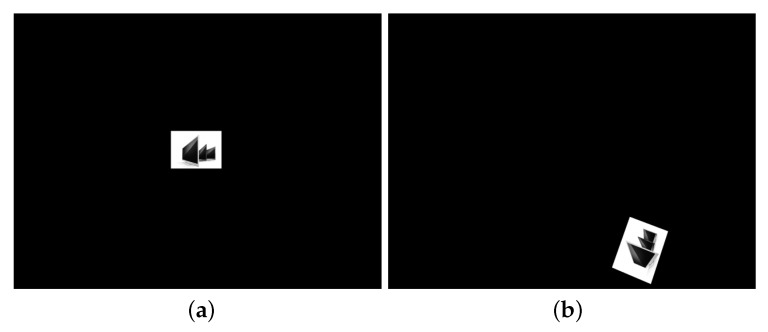
Results using conventional camera model: (**a**) the reference image and (**b**) example of the rotated images.

**Figure 4:**
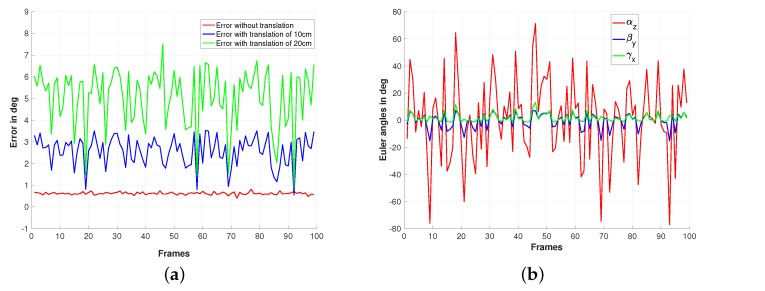
Simulation results using a perspective camera model: (**a**) Estimation error of the rotation and (**b**) rotation ground truth expressed.

The second simulation results are based on the same previous setup, but this time, it is a conventional perspective camera that is used to generate the images. As for the first simulations, we consider three sets of 100 images corresponding to transnational motions of different norms (0 cm, 10 cm, and 20 cm). Figure 3a,b shows the reference image and an example of the used rotated image for which the rotation has to be estimated. The ground truth of the rotational motions is shown on Figure 4b, while the accuracy of the rotation estimation is depicted on Figure 4a. From this Figure, it can be seen that the rotations are well estimated in the case where pure rotational motions are considered (refer to the red plot). It can also be noticed from the same figure (the blue and the green plots) that maximum rotation errors around $3.5^{\circ }$ and $7.5^{\circ }$ are observed respectively for the cases where the camera displacement involves a translational motion of norms equal to 10 cm and 20 cm, respectively.

### 3.2. Real Experiments

**Figure 5:**
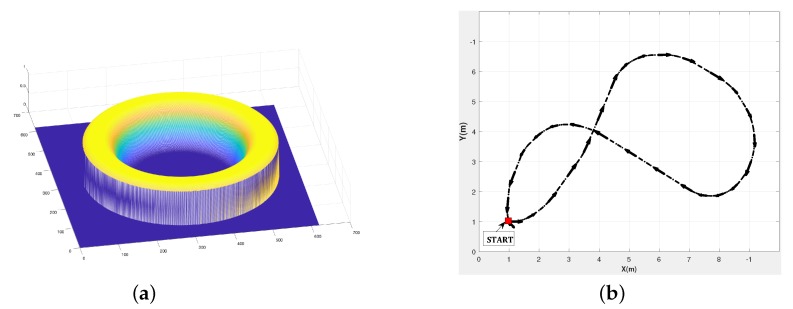
(**a**)Weight function and (**b**) Scenario 3: Eight trajectories of the pioneer robot.

The following experimental results use the OVMIS (Omnidirectional Vision Dataset of the MIS laboratory) dataset (http://mis.u-picardie.fr/~g-caron/pub/data/OVMIS_dataset.zip). Based on the same database, the preliminary work [23] has provided several experimental results where the proposed method is compared to the SPHARM (SPherical HARmonic analyses) method [15] and to the phase correlation (COR) and photometric (PHO) methods [29]. In the following, the proposed method is validated on a larger set of scenarios from the dataset. Moreover, we compare the performance of the proposed method using the spherical moments computed from the image intensities and from its gradient. We also test the effect of introducing the weighting function on the spherical moment computation of image acquired by catadioptric camera. The used weighting is a function of the pixel coordinates (x,y) of the following form:

(21) $$W\left(x,y\right)=e^{-\frac{{\left(r-r_{m}\right)}^{4}}{\sigma^{4}}}$$

where $r=\sqrt{{\left(x-u_{x}\right)}^{2}+{\left(y-u_{y}\right)}^{2}}$ is the distance of the pixel to the principal point $\left(u_{x},u_{y}\right)$ and σ is a scalar that tunes the wide of the image zone to be used. Note that W(x,y) has its maximum value 1 for $r=r_{m}$. Figure 5a shows the shape of the used weight function for this validation results. As can be seen from weighting function shape, the points close to the image border or to the blind zone in the mage center are given small weights to decrease the effect of appearance/disappearance of image parts. In all these experimental parts, the rotation motion is obtained by accumulating the estimated rotations between each two consecutive images.

The first validation result shown in the video (Supplementary Materials) is based on Dataset 1 of OVMIS with the Staubli robot arm. The considered sequence composed of 144 images has been acquired by applying regular pure rotations around the optical axis by a catadioptric camera mounted on the end effector of a Staubli in an indoor environment which corresponds to a rotation of $2.5^{\circ }$ between two consecutive images. First, the proposed method is tested by considering all the image sequences. Then, one image from 10 and from 20 are considered, which corresponds to rotations of $25^{\circ }$ and $50^{\circ }$, respectively, between tow consecutive images. As it can be seen from the video that the obtained results show that the proposed method estimates accurately the rotations in the three considered cases.

The second validation result shown as well in the video (Supplementary Materials) is based on the Scenario 1 of the Pioneer database acquired using a catadioptric camera. A similar setup to the previous case is used: a pure rotational motion around the z-axis in an indoor environment. The proposed method is tested by considering all the image sequences, one image from 10 and one from 20. The obtained results show again that the proposed method estimates accurately the rotations in the three considered cases.

The last experiment tests the proposed method on the more challenging Scenario 3 of the OVMIS dataset of a pioneer robot moving according to the trajectory described in Figure 5b. The achieved path is of length 25.27 m, in the form of 8 motions that include both rotational and translation motions. It has been achieved by the mobile robot in 160 s, which corresponds to 0.187 m/s average speed. The ground truth is obtained by incorporating gyroscopic corrections into the odometry data based on wheel encoders. From the video (Supplementary Materials), it can be seen that the mobile robot has traversed uneven terrain, with changing illumination conditions due to moving cast shadows of the trees and with appearance/disappearance of some image parts.

**Figure 6:**
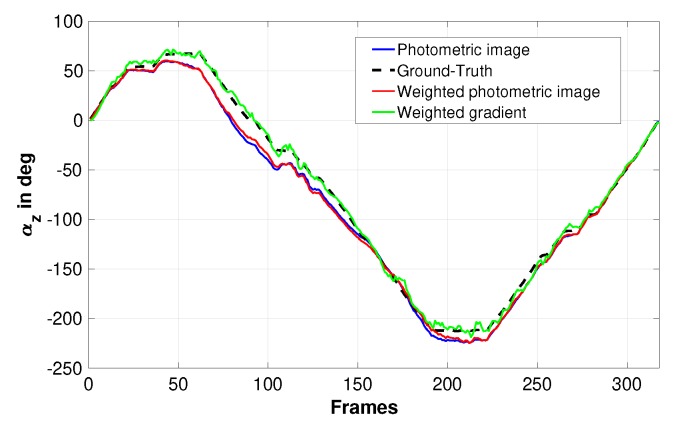
Estimated rotation of the pioneer robot around the Z-axis (Scenario 3).

Figure 6 compares the estimated rotation with/without using weighting function and based on moments computed from the image grey level or its gradient. From the obtained plots, it can be seen that using weighting function improves slightly the results obtained using the moment computed from the image intensities (compare the blue curve to the red one). On the other hand, it can be seen that the most accurate rotation estimation is obtained using the image gradient spherical moments and weighting function (refer to the green plot). The last experiments of the video (Supplementary Materials) describes the obtained results using the image gradient spherical moments by considering all the image sequences, one image from 10 and one from 20. From the obtained results, it can be seen that the rotation estimation is satisfactory for camera motion involving transnational and rotational motions.

### 3.3. Discussion

The triples computed from moment vectors provide a closed-form estimation to rotational motion. Compared to the classical method based on the alignment of principal axis, the proposed solution has no ambiguities on rotation formula. Recall that the alignment of principal axis method usually uses moments of higher orders to remove the ambiguities. If the estimated rotation is used as a feature in visual servoing or in active vision, the closed-form formula has a second advantage. For instance, as it has been proposed in References [30,31], the rotation vector is computed from the rotation matrix and used to control the rotational speed of a camera. For accurate visual servoing and active vision, most strategies are based on the analytical form of the visual features dynamics. Independently from the used method to estimate the rotation matrix, the relation between the time variation of a rotation vector and the rotational speed keeps the same form. On the other hand, its dynamic with respect to the translational speed becomes complex to obtain without a closed-form formula. Using the proposed solution in this paper, the dynamics of the rotation vector with respect to the whole degree can be obtained from those of the spherical moments [26,30,31].

A natural question arises about which are the best two triples to be used for building an orthonormal base. For instance, a base can be built from the two triples from $P_{1}$ to $P_{3}$ as well as by using two others from $P_{4}$ to $P_{7}$. Although it is well accepted that the sensitivity to noise increases with the moment orders, there is not much difference between results obtained using moments of orders 2 and 3 and those obtained using moments of orders 4 and 3. Moreover, an orthonormal base can be obtained using more than only two triples as it is achieved in Reference [31]. Finally, recall also that the rotation matrix can be obtained using all the triples $P_{i}$ as a generalized solution of the orthogonal Procrustes problem [32].

## 4. Conclusions

In this paper, a new method to estimate rotational motions using photometric moments has been provided. The obtained theoretical results have been validated using simulations and real experiments using onboard omnidirectional cameras and two different robots (robot arm and ground mobile robot). The proposed method has been also tested in different scenarios with appearance/disappearance of some image parts and lightening changes. Future works will be devoted to using photometric spherical moments for the control of the 6 degrees of freedom of a camera.

## Supplementary Materials

The following are available online at https://www.mdpi.com/1424-8220/19/22/4958/s1.

## Appendix A

• Triples computed from moments of order 2 and moments of order 3.

(A1) $$P_{1}=\left[\begin{array}{c} m_{003}m_{101}+m_{012}m_{110}+m_{021}m_{101}+m_{030}m_{110}+m_{101}m_{201}+m_{102}m_{200}+m_{110}m_{210}+m_{120}m_{200}+m_{200}m_{300}\\ m_{003}m_{011}+m_{011}m_{021}+m_{012}m_{020}+m_{020}m_{030}+m_{011}m_{201}+m_{102}m_{110}+m_{020}m_{210}+m_{110}m_{120}+m_{110}m_{300}\\ m_{002}m_{003}+m_{002}m_{021}+m_{011}m_{012}+m_{011}m_{030}+m_{002}m_{201}+m_{101}m_{102}+m_{011}m_{210}+m_{101}m_{120}+m_{101}m_{300} \end{array}\right]$$

(A2) $$P_{2}=\left[\begin{array}{c} m_{002}m_{120}-2m_{011}m_{111}+m_{020}m_{102}+m_{002}m_{300}-2m_{101}m_{201}+m_{102}m_{200}+m_{020}m_{300}-2m_{110}m_{210}+m_{120}m_{200}\\ m_{002}m_{030}-2m_{011}m_{021}+m_{012}m_{020}+m_{002}m_{210}+m_{012}m_{200}-2m_{101}m_{111}+m_{020}m_{210}+m_{030}m_{200}-2m_{110}m_{120}\\ m_{002}m_{021}+m_{003}m_{020}-2m_{011}m_{012}+m_{002}m_{201}+m_{003}m_{200}-2m_{101}m_{102}+m_{020}m_{201}+m_{021}m_{200}-2m_{110}m_{111} \end{array}\right]$$

(A3) $$P_{3}=\left[\begin{array}{c} m_{002}m_{102}+2m_{011}m_{111}+m_{020}m_{120}+2m_{101}m_{201}+2m_{110}m_{210}+m_{200}m_{300}\\ m_{002}m_{012}+2m_{011}m_{021}+m_{020}m_{030}+2m_{101}m_{111}+2m_{110}m_{120}+m_{200}m_{210}\\ m_{002}m_{003}+2m_{011}m_{012}+m_{020}m_{021}+2m_{101}m_{102}+2m_{110}m_{111}+m_{200}m_{201} \end{array}\right]$$
